# Supplementary material for: Single-Cell Sequencing Reveals the Regulatory Role of Maresin1 on Neutrophils during Septic Lung Injury
Source: Cells. 2022 Nov 23;11(23):3733. doi: 10.3390/cells11233733 (PMC9739442; doi:10.3390/cells11233733)
Supplement: Supplementary file 1 [file cells-11-03733-s001.zip › cells-1978045-supplementary.pdf]

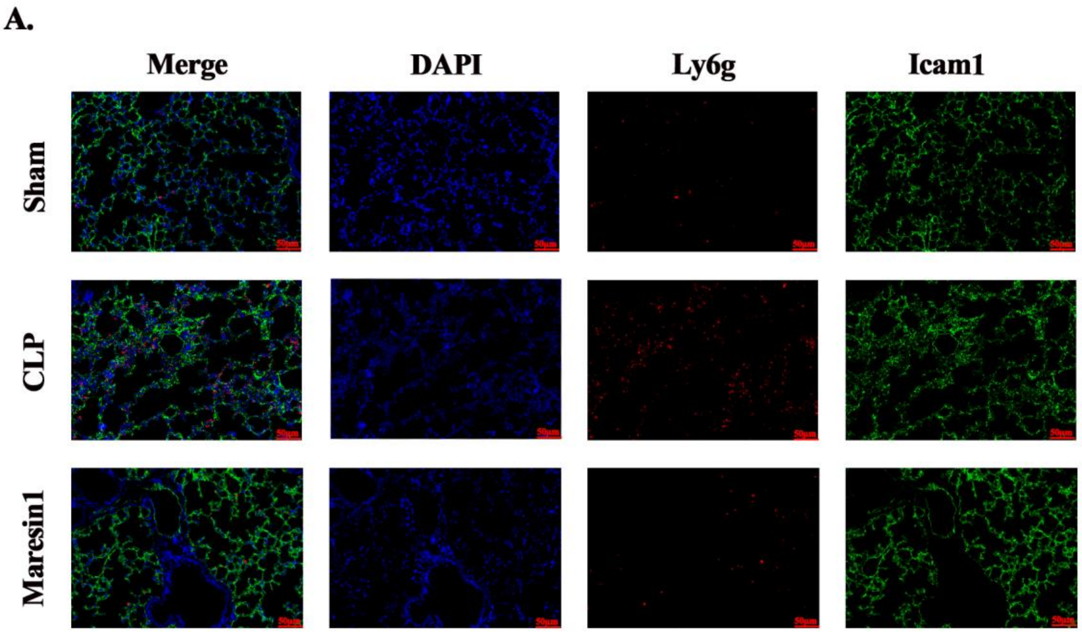

Figure S1 : The double staining immunofluorescence of Ly6g and Icam1 of the lung tissue.

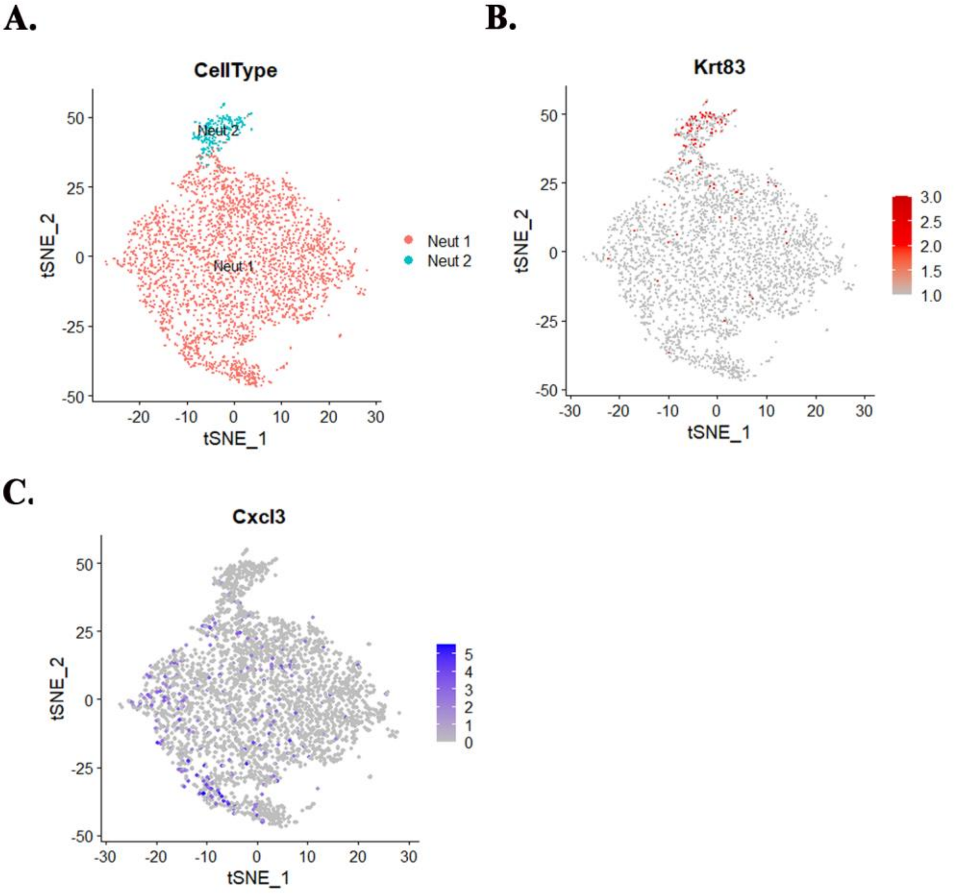

Figure S2 : The existence of Neutrophil3-Cxcl3 subpopulatio was confirmed in the public data set.
